# Highlights of the XXI annual meeting of the Brazilian Society of Protozoology, the XXXII annual meeting on Basic Research in Chagas' disease & an international symposium on vesicle trafficking in parasitic Protozoa – 7 to 9 November 2005, Caxambu, Minas Gerais, Brazil

**DOI:** 10.1186/1475-9292-5-4

**Published:** 2006-08-17

**Authors:** Jeffrey Shaw, Sergio Schenkman, Mauricio Martins Rodrigues

**Affiliations:** 1 Departamento de Parasitologia, Instituto de Ciências Biomédicas, Universidade de São Paulo, Av. Prof. Lineu Prestes, 1374, 05508-900 São Paulo, Brazil; 2 Departamento de Microbiologia, Imunologia e Parasitologia, Universidade Federal de São Paulo, R. Botucatu 862 8^A^, 04023-062 São Paulo, Brazil; 3 Departamento de Microbiologia, Imunologia e Parasitologia and Centro Interdisciplinar de Terapia Gênica (CINTERGEN), R. Mirassol, 207, 04044-010, São Paulo, Brazil

## Abstract

This report focuses on the 2005 Annual meeting held in Caxambu, Minas Gerais, Brazil that was convened and organized by the Brazilian Society of Protozoology . This is an annual event and details of these meetings can be found on the Society's website. Within the space available it has been impossible to cover all the important and fascinating contributions and what is presented are our personal views of the meetings scientific highlights and new developments. The contents undoubtedly reflect each author's scientific interests and expertise. Fuller details of the round tables, seminars and posters can be consulted on line at .

## Background

The meeting was attended by principally Brazilian scientists and students and a smaller number participants from other Latin American countries, Europe, the United States and Japan. Of the 546 participants 72.94% (380) were students. The scientific sessions were divided into 10 peer lectures, 13 round tables in which 4 invited speakers explored specific areas and 382 posters. There were also 4 sessions in which selected posters were presented orally. In these sessions a total of 35 posters were presented by the senior author who in most cases was a postgraduate student. In this report we provide a "snapshot" of what, in our view, are the highlights of newest developments.

The event was inaugurated by José Franco da Silveira (vice president of the Brazilian Society of Protozoology) who presented an autobiographical sketch of the 2005 recipient of the Samuel Pessoa award – Prof. Luiz Hildebrando Pereira da Silva. Prof. Hildebrando, director of the "Instituto de Pesquisas em Patologias Tropicais de Rondônia" (IPEPATRO), gave the opening Samuel Pessoa lecture emphasizing that parasitological research projects in a Tropical Research Institute in Amazonia integrate science and regional health problems through collaborative programs and "brain circulation". He highlighted the economic disequilibrium in Brazil caused by the destruction of the Amazonian forest by logging companies, cattle farmers and soja bean planters. In spite of these products being some of the countries principal exports they produced an unacceptably low salary index per capita/per acre which could be higher if native forest resources were exploited intelligently.

## Cellular traffic

Mark Field (Cambridge University, UK) presented his recent data on the developmental regulation of endocytosis in *Trypanosoma brucei*, which undergoes massive remodeling of its surface. He showed that bloodstream and procyclics have different levels of small GTPases (Rabs) that control the traffic. Rab 11 is 20 fold enriched in bloodstream compared to procyclic forms, while Rab 4 levels are similar. Using RNAi his group demonstrated that these two different stages have different patterns of cellular traffic pathways and control and that over 600 genes are involved in trafficking. But how is the regulation of genes orchestrated and what are mechanisms that control the differential traffic during development? Ulisses Lopes from UFRJ showed that the Rho proteins of *Trypanosoma cruzi *are small GTPases responsible for cytoskeleton remodeling mainly through actin filaments. One of them, TcRho1 presents a CaaX motif at the C-terminus that targets the protein for farnesylation. Mutants lacking the CaaX motif results in cell dispersion and death during differentiation, suggesting that is required for metacyclogenesis. He also identified the OCRL1 and TcRJLs. The first is responsible for Lowe Syndrome in humans but its function in trypanosomes is unknown. The second belongs to a new family of Ras-related GTPase proteins in *T. cruzi *genome and is found in other trypanosomes and *Giardia*.

Using electron microscopy Maurilio Soare's group (UFRJ, Brazil) studied the endo and exocytic vesicles near the flagellar pocket of *Trypanosoma cruzi *and presented evidence of the presence of GM1 enriched lipid rafts in these entry points. This type of membrane structure was also detected in the flagellum of *T. brucei *and *T. cruzi *by David Engman's group (Northwestern University, Chicago, USA). He showed that the flagellar calcium binding protein (FCaBP) in these trypanosomes is dependent on myristoylation and palmitoylation, and calcium binding acts as a switch for the membrane localization. This is part of a kinase signaling pathway that controls flagellar function by affecting the lipid order and membrane organization. Lipid raftsstructures were also found by Narcisa Cunha e Silva's team (UFRJ, Brazil) in the reservosomes, which are large organelles present in *T. cruzi *epimastigotes that accumulate proteins and lipids. When the parasite undergoes metacyclogenesis the reservosomes disappear.

The diversity and peculiarity of Rab small GTPase in vesicular trafficking of ameba was presented by Tomoyoshi Nosaki (Gunna University Graduate School of Medicine, Japan). He compared phagocytosis of pathogenic *Entamoebae histolytica *with non-pathogenic *Entamoebae dispar*. Amoeba phagosomes show small GTPases, Rab1/5/7/8 and 11 in addition to several cytoskeleton components, signal transduction, transport, mitochondria-like protein, metabolic enzymes, ubiquitin systems and several hydrolases (Euk. Cell and MBP 2005). Interestingly, amoebae have a larger number (91) and unique Rab GTPase compared to humans or other higher eukaryotic cells (~60). Moreover, they have a unique pre-phagosomal vacuole (PPV), which contains protease and amoebapore that fuse to form primary and secondary phagosomes. Nosaki's group characterized proteins interacting with Rab5 and Rab7, involved in the formation of PPV.

Isabelle Coppens (John Hopkins University School of Public Health, USA) showed that *Toxoplasma gondii *uses cellular trafficking to obtain host cell nutrients. The parasite manipulates the distribution of host cell microtubules and vimentin but not actin to surround the parasitophorous vacuole (PV) in a way that a corset of host microtubules is formed around it. This dynamic association is used to divert low-density lipoproteins (LDL). One components of this machinery is GRA7, a 236 amino acid protein, which acts like a garrote that sequesters host endocytic organelles within the vacuolar space. My-Hang Huynh's team (John Hopkins University, USA), presented aspects of trafficking involved in *T. gondii *cell invasion in which proteins found in micronemes are secreted to form the new PV. By knocking-out *T. gondii *M2AP protein, they found a decrease in the microneme MIC2 expression, which is retained in the ER/Golgi, severe impairment of cell invasion in vitro, attenuated virulence as well as gliding motility problems. They propose as a model in which M2AP forms a complex with MIC2 in an early endosome, which is processed and added to micronemes.

## Host parasite interaction

Oscar N. Mesquita (UFMG, Brazil) examined membrane alterations during the interaction of macrophages with *Leishmania *by defocusing under optical microscopy. He detected and quantified small random changes and large alterations as ruffles on the cell surface. The variations were conserved, indicating that they are produced by discrete events. Ruffles were inhibited by adding cytochalasin. When a parasite is added to the cell using the optical tweezers he found dramatic perturbations in the small movements, but ruffling activity was not modified. Antonio Gonzalez Aguilar (CSIP, Spain) demonstrated that the alternative splice of the Lyt1 gene of *T. cruzi *generates a membrane protein. This membrane protein is the TcTox involved in the parasite's escape from the PV. *Phytomonas serpens *that expresses this gene is able to escape from the PV and multiply in the macrophage's cytoplasm. The other spliced form of Lyt1, codes for a protein expressed in the epimastigote's kinetoplast, and is probably involved in DNA replication.

## Control of gene expression

Artur Scherf (Institute Pasteur, France) described recent advances in explaining the expression of Var genes by *Plasmodium falciparum*. Var genes encode the *Plasmodium *erythrocyte surface proteins (Pefr1), whose expression delimits infected red cell adhesion. Dr Scherf proposed a model for the control of Car gene expression that depends on where the Var gene is located in the nucleus. Non-expressed Var genes are in heterochromatin regions that prevents transcription. In contrast, the expressed Var gene chromatin is acetylated outside the heterochromatic region. The next question to be answered is how the gene's expression site is selected?

*T. cruzi *surface mucin-like molecules are encoded by a large multigene family. Vanina Campo (Instituto de Investiagaciones Biotecnológicas, Argentina), explained that the expression of this diverse group of genes is controlled by post-transcriptional mechanism, including alternative splicing and mRNA stabilization. Key factors that control expression are RNA recognition motives in these genes, which are sites for binding of protein factors that stabilize specifically groups of mRNA. Stenio Fragoso (IBMP, Brazil) characterized TcZFP1a CCH-zinc finger protein, possibly involved in post-translation control of gene expression. He showed that it is expressed cells submitted to nutritional stress and is located in cytoplasmic spots. The protein expressed in *E. coli *in fusion with GFP and purified by Ni-columns interacts with RNA sequences containing poly C RNA. It does not bind to AU rich elements (ARE). This, together with other CCH zinc finger proteins, is differentially expressed during metacyclogenesis as seen by microarray analysis.

*T. cruzi *is composed by two major phylogenetic groups, *T. cruzi *I and *T. cruzi *II. Santuza Teixeira's group (UFMG, Brazil) found that multigene families of group I are more homogeneous than those of group II. Hybrid strains showed intermediate variability. According to her data, mismatch excision repair may be different in the two groups, particularly TcMSH2 activity, one of the proteins responsible for this type of repair. Nancy Sturm (UCLA, USA) showed polymorphisms in the kinetoplast maxicircle sequences of these two groups and that a variable RNA editing could compensate these differences.

A key factor in explaining differential gene expression of trypanosomatids is the control of translation initiation. The group lead by Oswaldo Pumpilio Melo Neto (FIOCRUZ, Brazil) presented results characterizing multiple homologues for the eIF4E subunits of *Leishmania *and *Trypanosoma *involved in cap binding and translation initiation control in trypanosomes. *L. major *has four homologues but only eIF4E1 binds to cap, while in *T. brucei *both eIF4E1 and E2 are involved in this same process but that, only eIF4E3 was essential.

## Epidemiology

The Sudan is one of the countries that has had some of the worst epidemics of visceral leishmaniasis (VL) known to man and Muntaser Irahim (Institute of Endemic Diseases, Sudan) compared this disease to a serial killer. The actual number of people who have died over the years is unknown but when the epidemic in the West Nile region was first recorded in the 1950's a whole tribe died was wiped out. Since then there have been estimates that over 250,000 people have been victims of the "serial killer". The variable severity of the disease is considered to be linked to both ethnic and geographical elements. Recently it has been shown that the graver symptoms leading to death are linked to certain genes. In Brazil it has been noted that VL epidemics have a 10 year cycle and it was a surprise to learn that in the Sudan the disease also strikes every ten years. Detailed molecular studies have shown that the aetiological agent, *Leishmania *(*Leishmania*) *archibaldi *is closer to *L.*(*L*.) *donovani *and it has been suggested that the latter originated from the former when man migrated from Africa. There is a possibility that there are wild animal reservoirs and that these are in fact the primary source, however, in its epidemic form the disease is clearly an anthroponosis.

## Highlights of immunology

Diverse aspects of immunity and immunopathology of malaria, leishmaniasis, Chagas' disease and toxoplasmosis were addressed.

The malaria speakers enriched our knowledge on diverse topics such as the molecular aspects of the immunopathology of *P. falciparum *malaria and the presence of cross-reactive epitopes between diverse species of malaria parasites. Jürg Gysin (Institut Pasteur, France) described his work on a roptry protein (RSP-2) which is present on the surface of non-infected human erythrocytes. His results suggest that in part anemia is related to the destruction of these cells by RSP-2 specific antibodies with the aid of monocytes. Subsequently, Lars Hviid (Rigshospitalet, Denmark) summarized several years of studies on pregnancy-associated malaria (PAM) which established that PAM resulted from preferential sequestration of parasitized red blood in the placenta. The molecular target for immunity against PAM was identified as the PfEMP-1 (*var*2^CSA^gene) which is expressed on the surface of infected red blood cells capable of binding to chondroitin sulfate A. Based on this they proposed an anti-PAM vaccine. Gerhard Wunderlich (USP, Brazil) concluded that the repertoire of the *P. falciparum var *genes in the Amazon Basin is limited and that this indicated a reduced gene pool of this species throughout the region. Denise Mattei (Institute Pasteur, France) elucidated some of the mechanism by which *clag-2 *gene products modulates adhesion of infected red blood cells through expression of certain PfEMP-1. Finally, Antoniana Krettli (FIOCRUZ, Brazil) discussed the presence of cross-reactive epitopes present in sporozooites of *P. falciparum *and *P. gallinaceum *sporozooites. This cross-reactive epitope may be a new antigenic marker for the detection of protective immunity against *P. falciparum *sporozooites.

Flavia Ribeiro-Gomez (UFRJ, Brazil) found that the course of experimental infection with *L. major *can be modified by the presence of apoptotic neutrophils. In the case of BALB/c mice these cells have a deleterious role in the infection mediated by the increase in secretion of TGF-beta by macrophages. On the other hand, the presences of apoptotic neutrophils increase the resistance of C57BL/6 mice by a mechanism that is dependent on the neutrophil elastase, and TNF-alpha and NO secretion.

The presentations on the immunity to *T. cruzi *focused on subjects that are expected to be the new trends in the years to come. Sarah Williams-Blangero (South-Western Foundation for Biomedical Research, USA) described elegant human genetic studies concluding that several clinical features of chronic Chagas' disease are genetically linked. Nobuko Yoshida (UNIFESP, Brazil) shed some light on the poorly understood molecular mechanisms responsible for oral infection, describing also a possible method for the eventual blockage of this type of infection. Maurico Rodrigues (UNIFESP, Brazil) focused his presentation on a so far poorly studied molecule extremely abundant in the amastigote forms of the parasite. He concluded that the amastigote surface protein-2 is highly conserved among the different strains of the parasite being recognized by protective CD4 Th1 and CD8 Tc1 cells. These results indicate that this molecule may provide an excellent target for vaccination. Walderez Dutra (UFMG, Brazil) explored the presence of regulatory molecules on the surface of antigen presenting cells (APC) as key factors controlling the reactivity of pathogenic T lymphocytes. So far, she has observed that APC from symptomatic individuals have an activated profile that may explain at least in part the higher reactivity of T lymphocytes. Studies in Chagasic humans performed by Juliana de Assis (FIOCRUZ, Brazil) described an interesting correlation between the frequency of CD4+CD25+ regulatory cells and the indeterminate forms of the disease. Joseli Lannes (FIOCRUZ, Brazil) presented data where she described that CCR5 inhibitors may be explored as tools to reduce the heart immunopathology of experimental Chagas' Disease.

## Monoxenic Trypanosomatids

The presentations at round table on the monoxenics and evolutionary perspectives of the Trypanosomatidae family ranged from the origin of endosymbionts to origin of the group. Cristina Motta (UFRJ, Brasil) emphasized that the trypanosomatid endosymbiont cannot survive outside its host and that like mitochondria it lacks the septum and FtsZ ring that are essential in bacterial division. Recent sequence data indicates that they are closely related to *Bordetella bronchiseptica *and that endosymbiosis was a single event. Based on the 18S rRNA, SL RNA and protein coding genes from an extensive taxa set Julius Lukes (Institute of Parasitology, Czech Republic) concluded the trypanosomatids were monophyletic group that originated from paraphyletic bodonids. The 18S rRNA was not informative for insect parasites and indicated that members of this group are not host specific. Similarly bird trypanosomes do not appear to be vector specific and are found in mosquitoes, black flies, hippoboscids and midges. The vector of the *T. avium *clade in the Czech Republic appears to be black flies while the United Kingdom the vectors of the *T. corvi *clade are hippoboscids and mosquitoes. There appears to be an overall low level of host specificity with the surprising possibility of insect parasites infecting warm blooded vertebrates. The available taxonomic data strongly favours the hypothesis that the group evolved from flagellates of insects. One of the important biological characters of the trypanosomatids is that their morphology is reversible during their life cycles and Angela Lopes (UFRJ) questioned what mechanisms are responsible for this cellular modulation. She drew attention to potential importance of platelet-activating factor (PAF) in this process. PAF is a phosopholipid with that has diverse physiological and patho-physiological actions. Another substance found in serum and reduviid bug saliva, lysophosphatidycholine (LPC) has been found to modulate cellular differentiation of *Herpetosoma samuelpessoai *and it is possible that LPC reacts via the PAF receptor. There has been an increase in immuno-suppressed individuals in recent years and human cases of monoxenic trypanosomatids in man have been recorded. With this in mind Elvira Saraiva (UFRJ, Brazil) investigated the infectivity of *Blastocrithidia culcis *to human monocytes that had been exposed to HIV-1 transactivator protein. The intracellular survival of *B. culcis *was greater in cells exposed to HIV-1 Tat protein and TGF-β1. In *Aedes aegypti *the parasite adhered and penetrated the gut wall and adhered to the salivary gland wall. This could lead to transmission by bite which could lead to infection in immuno-suppressed individuals.

## Chemotherapy & treatment

The problems of developing new drugs for neglected diseases were discussed by Simon Croft, coordinator of Drugs for Neglected Diseases initiative (DNDi – ). The development cost of a new drug is in the order of US$800 million dollars which is prohibitive for neglected diseases such as malaria and tuberculosis. He emphasized that there have been enormous advances in biochemical and cell biological studies of the pathogens such as leishmania and trypanosomes but that the data must be used in an organized fashion for the Research and Development (R & D) of new drugs. The major R & D stumbling block in the past has been the lack of coordinated efforts between commercial companies who have the expertise and groups from the countries in which the diseases are endemic. However this is possible with sustainable funding and to this end non profit making organizations have been formed to funnel funds into this area and forge collaborative links. A virtual model targeting the product profile has been developed to accompany development at different stages so that decisions as to the feasibility of success can be critically assessed, thus avoiding investing in a product that will not meet the basic requirements.

Other participants discussed the specific need for the development of drugs for leishmaniasis and American trypanosomiasis. Eder Romero (Universidade Nacional de Quilmes, Argentina) explored the use of nanotechnology for delivering drugs in concentrations up to 100 times less than those administered by traditional methods. The delivery vehicle (nanoship) also has the advantage of being able to target a specific disease compartment at either the tissue or cellular level. Federico Gomes-de-las-Herus, (GlaxoSmithkline disease of the developing World Drug Discovery Centre, Spain) explained how the GSK centre, which has 100 full time scientists and the full support of the parent drug company, works via a partnership with Medicines for Malarial Venture (MMV) and Global Alliance for TB (GATB) in developing new drugs for malaria and tuberculosis. An important concept in this process is the Target Product Profile which for malaria is: treatment of uncomplicated malaria, suitable for sensitive & resistant *Plasmodium*, oral administration and fast activity, 3 day treatment, well tolerated, suitable for use in combinations and affordable for the target population. It was emphasized that the high cost of drugs is because very few reach the market place. Hector Freilij (Hospital de Niños Ricardo Gutiérez, Argentina) drew attention to the importance of congenital transmission of Chagas disease in urban areas of large towns such as Buenos Aires. The infected mothers are from endemic areas and are asymptomatic. Of three children only one may become infected during pregnancy and treatment of the children under 5 years old is successful. In urban areas of Argentina Chagas disease has become a childhood disease and the majority of the children are asymptomatic. A major question is if such cases go untreated will they develop the more severe clinical forms of Chagas disease later in their lives. Luis Villa Villanueva (Médecins San Frontiéres, Spain) voiced his concern as to the availability of drugs for the treatment of Chagas Disease since the major drug companies were not interested in continuing to manufacture them. Alternative manufacturing sources within endemic countries are a solution to this problem and Biomaguinhos (FIOCRUZ, Brazil) has signed contracts to produce some. It was pointed out that this may solve Brazil's future problem but will it help other Latin American countries where Chagas disease is endemic?

The treatment of the extreme variety of clinical forms of cutaneous leishmaniasis that may lead to more severe clinical symptoms such as mucocutaneous leishmaniasis and diffuse cutaneous leishmaniasis is a challenge to the clinician. Edgar de Carvalho (UFB, Brasil) addressed this problem by documenting some of the important immunological parameters (IFN-γ, TNF-α, IL2, IL-10 IL15, and T cells CD4, CD8, CD69, CD28^-^, CD62L^-^) associated with the different clinical forms. With the intuitive of improving the chemotherapeutic responses GM-CSF and pentoxyfilline were associated with antimony therapy to control some of the adverse effects caused by the cytokines.

## Parasite vector interaction

Marcelo Jacobs-Lorena (Malaria Research Institute, USA) reviewed the approach of using transgenic mosquitoes in preventing malaria transmission. The main strategy has been to genetically engineer mosquitoes to make them resistant to the pathogen, introduce them into the population that will theoretically reduce transmission. He stressed that this is a complementary approach to drugs and vaccines. Due to the complexity of the fitness problem in the nature, his group is now trying to introduce transformed bacteria as a source of heterologous genes (paratransgenic). He also showed his recent results describing the role of SPRN6, a member of the serine protease inhibitor (serpin) family, which is expressed by insect infected cells. SPRN6 RNAi and knockouts have an increased infection load. More interesting is the fact that expression of this protein is differently involved in the mosquitos' defenses against different species of *Plasmodium*. In another presentation, Eloi Garcia (FIOCRUZ, Brazil) reported the role of eicosanoids in trypanosomatid-triatomine interactions. He focused his talk on the interaction of *Rhodnius prolixus *with *Trypanosoma rangeli*. The vector is infected when it feeds; the parasite proliferates in the gut and is eliminated with faeces but in approximately 10% of the insects the flagellates penetrate the gut wall and reach the salivary gland. Data published in 2004 showed that eicosanoids produced by insect cells protect the vector against *T. rangeli *induced mortality. The increase in mortality is correlated with decrease in aggregation of hemocytes, the defense cells that are present in the insect hemolymph. In contrast, hemocyte microaggregation is promoted by arachidonic acid, which protects the insects. Possible platelet-activating factor (PAF) like molecules are being proposed to mediate these aggregation effects since the WEB 2086, a PAF agonist, increased insect survival, at the same time that less hemocytes aggregation was seen.

The meeting's closing conference was given by Antonio Teixeira (UnB, Brazil) who discussed the pathology of Chagas Disease and evidence that *T. cruzi *is in fact the genetic vector of kinetoplast DNA. The integration of parasite kDNA into the vertebrate genome in this scenario would be responsible for the immunopathology of Chagas' disease.

## Competing interests

The author(s) declare that they have no competing interests.

## Authors' contributions

All authors contributed equally to the preparation of this report.

